# The Impact of High-Intensity Interval Training on Cardiometabolic, Neurologic, Oncologic, and Pain-Related Outcomes: A Comprehensive Review of Systematic Reviews

**DOI:** 10.3390/jcm14238328

**Published:** 2025-11-24

**Authors:** Dmitriy Viderman, Yeltay Rakhmanov, Mina Aubakirova, Sultan Kalikanov, Michael Fredericson

**Affiliations:** 1 School of Medicine, Nazarbayev University, 5/1 Kerey and Zhanibek Khans Ave., Astana 02000, Kazakhstan; 2 Department of Orthopaedic Surgery, Division of Physical Medicine & Rehabilitation, Stanford University, 450 Broadway, Redwood City, CA 94063, USA; mfred2@stanford.edu

**Keywords:** high-intensity interval training, exercise, physical activity, health, outcomes

## Abstract

High-intensity interval training (HIIT) has gained attention for its potential to improve health outcomes across various conditions. Thus, the aim of the study was to summarize studies on HIIT to understand its effects on various health outcomes. We conducted an umbrella review of systematic reviews and meta-analyses. PubMed, Cochrane Database of Systematic Reviews, EMBASE, Scopus, CINAHL, and Web of Science were searched for relevant articles. The experimental group was subjected to HIIT with or without treatment, while the control group comprised individuals who underwent alternative forms of training or were non-exercisers. Included studies were systematically analyzed for effects of HIIT and cardiovascular, respiratory, metabolic, neurological, gastrointestinal, immunological, and survival-related outcomes. Of 336 identified systematic reviews, 133 were included in the final analysis. HIIT was found to confer significant physiological benefits, including improvements in body composition, cardiovascular and metabolic parameters, and mental health outcomes. Studies demonstrated the efficacy of HIIT across diverse patient populations, with comparable or superior effects to moderate-intensity continuous training in conditions such as diabetes, cardiovascular diseases, neurological, oncologic, and pain-related disorders. Our review highlights the potential of HIIT as a time-efficient intervention for improving health outcomes and managing chronic diseases. However, interpretation of the results should be performed cautiously due to the heterogeneity observed. High-intensity interval training shows promise as an effective strategy for managing chronic diseases among diverse patient populations. Future research should focus on refining HIIT protocols and elucidating their long-term effects and sustainability.

## 1. Introduction

Currently, chronic diseases contribute to almost half of the total global disease burden; estimates suggest that six out of every ten deaths are associated with chronic conditions. Studies have highlighted the importance of consistent physical activity in lowering the risk of coronary heart disease, stroke, diabetes, hypertension, colon cancer, breast cancer, and depression. The prevalence of chronic diseases highlights the need for preventive actions like physical exercise as a primary solution to maintain or improve health [1]. People without health problems should have at least 2.5 h of physical exercise of moderate intensity or 1.15 h of exercise of high intensity weekly to keep or strengthen their health, as per recommendations by the World Health Organization and the American College of Sports Medicine [1,2]. High-intensity interval training (HIIT) involves a cycle of brief, high-intensity physical exercise periods alternating with low-intensity recovery periods. The feasibility of HIIT as a substitute for the usual physical activity approaches has been previously investigated [2,3]. HIIT provides comparable or superior health benefits to traditional exercise approaches, including increased aerobic capacity and reduced risk factors for many diseases and conditions [2,3,4]. Specifically, HIIT has been found to have significant physiological benefits, such as improving oxygen uptake, body composition, blood glucose and pressure levels, inflammatory markers, exercise capacity, cognitive and mental health, and quality of life [2,4]. HIIT has been suggested as a potential solution to the everyday barriers preventing people from exercising, particularly the inability to devote much time to the activity. Compared with traditional exercise approaches, HIIT provides an opportunity to improve health while spending less time. This makes it an attractive option for those who struggle to find time for physical activity. HIIT is particularly beneficial for improvements in aerobic capacity and minimizing the risk of adverse conditions potentially leading to metabolic problems, such as hypertension and insensitivity to insulin [2,5]. A number of systematic reviews have emerged in broader clinical contexts, but they were limited in scope to selected populations or outcome categories. These include oncologic, neurological, musculoskeletal, and pain-related conditions, as well as pediatric and older adult groups. No comprehensive synthesis has integrated these diverse findings to identify consistent patterns and evidence gaps across various health outcomes. Therefore, this umbrella review provides an updated, cross-disciplinary synthesis of the effects of HIIT on multiple physiological systems, summarizes its comparative efficacy versus moderate-intensity continuous training, and highlights fields that require further investigation and clinical standardization. Specifically, the objectives were to (a) collect and examine systematic reviews focused on the effects of HIIT to understand its outcome values and (b) synthesize cumulative effects of HIIT across different health domains and patient populations, based on evidence from systematic reviews and meta-analyses.

## 2. Materials and Methods

### 2.1. Criteria for Inclusion

We used the following inclusion criteria for this review: (1) study design; (2) patient population; (3) intervention/control; and (4) outcomes.

Patient population: We selected participants without restriction to gender, age, diagnosis, comorbidities, health, and physical activity status.

Intervention: The experiment group received HIIT (all types without limitation to any specific type of HIIT) with or without standard treatment.

Control: Other types of training, such as moderate- or low-intensity continuous training or non-exercising population.

Outcomes: The primary outcomes of this review included:

- Cardiometabolic outcomes;
- Neurological outcomes;
- Metabolic/endocrine outcomes;
- Oncologic outcomes;
- Pain-related outcomes.

Study design: Systematic reviews with or without meta-analyses.

### 2.2. Criteria for Exclusion

We used the following inclusion criteria for this review: (1) study design; (2) population; (3) intervention; (4) controls; (5) outcomes; (6) availability; and (7) language.

Study design: Commentaries, editorials, letters, protocols, primary studies (randomized controlled study or observational study), conference abstracts without full text, or older versions of the same systematic review.

Population: Non-human (animal or in vitro) studies or populations not involving human participants.

Intervention: Reviews without a HIIT (i.e., only moderate/low-intensity training or non-exercise).

Controls: Any control, excluding only if HIIT is not clearly defined.

Outcomes: Reviews that do not clearly report primary outcome (cardiometabolic, neurological, metabolic/endocrine, oncologic, or pain-related).

Availability: In the case that the full text is unavailable after reasonable efforts to obtain it.

Language: In the case that the full text is not available in English after reasonable efforts to obtain it.

### 2.3. Search Strategy

The list of databases searched is as follows: PubMed, Cochrane Database of Systematic Reviews, EMBASE, Scopus, and Web of Science. The search terms used were: “high intensity interval training” OR (“high intensity” AND “interval” AND “training”) OR “high intensity interval training” OR (“high” AND “intensity “AND “interval” AND “training”) OR (“high intensity interval training”) AND (“insurance benefits” (“insurance” AND “benefits”) OR “insurance benefits” OR (“health” AND “benefits”) OR “health benefits”). We manually screened the reference sections of the SRs and MAs. We contacted the corresponding authors for further explanations if deemed necessary. Three authors worked independently utilizing the pre-determined search strategy, and any cases of mismatch were further resolved. Two reviewers performed data extraction.

### 2.4. Data Synthesis and Analysis

We categorized the type of objectives of the studies, diseases and comorbidities studied, effects of HIIT, and outcomes. We designed “evidence tables” to facilitate an in-depth description of the included SRs and the respective findings.

### 2.5. Methodological Quality Assessment

The quality appraisal was performed by AMSTAR-2 (A MeaSurement Tool to Assess systematic Reviews) [6]. The appraisal was performed by two authors independently and compared with each other. Discrepancies, if any, were resolved by discussion. Each item was rated as “Yes” (meets the standard), “No” (does not meet the standard or unclear), “Partial yes” (meets the standard with some limitations), and “N/A” (not applicable because the study did not perform the quantitative synthesis).

Overall confidence was determined based on the absence and presence of critical domains (items 2, 4, 7, 9, 11, 13, 15). The rating is as follows:

- High: 0–1 non-critical weakness;
- Moderate: more than one non-critical weakness;
- Low: 1 critical flaw (with/without other weaknesses);
- Critically low: more than 1 critical flaw (with/without other weaknesses).

## 3. Results

### 3.1. Included Studies

We initially identified 336 SRs matching the inclusion criteria, 133 of them were finally selected [2,7,8,9,10,11,12,13,14,15,16,17,18,19,20,21,22,23,24,25,26,27,28,29,30,31,32,33,34,35,36,37,38,39,40,41,42,43,44,45,46,47,48,49,50,51,52,53,54,55,56,57,58,59,60,61,62,63,64,65,66,67,68,69,70,71,72,73,74,75,76,77,78,79,80,81,82,83,84,85,86,87,88,89,90,91,92,93,94,95,96,97,98,99,100,101,102,103,104,105,106,107,108,109,110,111,112,113,114,115,116,117,118,119,120,121,122,123,124,125,126,127,128,129,130,131,132,133,134,135,136,137,138].

#### 3.1.1. Patient Population and Objectives of the Meta-Analyses

The effects of HIIT have been studied in a wide variety of diseases and conditions, including cardiovascular, respiratory, metabolic, oncologic, neurologic, and musculoskeletal diseases. Many studies focused on the effects of HIIT on several organ systems (cardiovascular, pulmonary, and endocrine/metabolic) and diseases. The objectives of the included SRs and MAs studied the following effects of HIIT (Table 1) [2,7,8,9,10,11,12,13,14,15,16,17,18,19,20,21,22,23,24,25,26,27,28,29,30,31,32,33,34,35,36,37,38,39,40,41,42,43,44,45,46,47,48,49,50,51,52,53,54,55,56,57,58,59,60,61,62,63,64,65,66,67,68,69,70,71,72,73,74,75,76,77,78,79,80,81,82,83,84,85,86,87,88,89,90,91,92,93,94,95,96,97,98,99,100,101,102,103,104,105,106,107,108,109,110,111,112,113,114,115,116,117,118,119,120,121,122,123,124,125,126,127,128,129,130,131,132,133,134,135,136,137,138].

#### 3.1.2. Reported Diagnoses

The effects of HIIT were studied in patients with the following conditions: diabetes mellitus type 1 (DMT1) and 2 (DMT2), obesity, coronary artery disease (CAD), myocardial infarction (MI), post-MI syndrome, heart transplant, cancer, chronic heart failure (CHF), coronary artery bypass surgery (CABG), arrhythmias, stroke, atherosclerosis, prehypertension, hypertension (HTN), asthma, chronic obstructive pulmonary disease (COPD), Parkinson’s disease, muscular skeletal (MSK) disorders (non-specified), metabolic syndrome, polycystic ovary syndrome (PCOS), schizophrenia, nonalcoholic fatty liver disease (NAFLD), spinal cord injury, cancer (lung, breast, bladder, rectal, liver, rectal, testicular), Alzheimer’s disease, mental illness, anxiety, eating, stress disorders [7,8,9,10,11,12,13,14,15,16,17,18,19,20,21,22,23,24,25,26,27,28,29,30,31,32,33,34,35,36,37,38,39,40,41,42,43,44,45,46,47,48,49,50,51,52,53,54,55,56,57,58,59,60,61,62,63,64,65,66,67,68,69,70,71,72,73,74,75,76,77,78,79,80,81,82,83,84,85,86,87,88,89,90,91,92,93,94,95,96,97,98,99,100,101,102,103,104,105,106,107,108,109,110,111,112,113,114,115,116,117,118,119,120,121,122,123,124,125,126,127,128,129,130,131,132,133,134,135,136,137,138].

### 3.2. Findings

**Figure 1:**
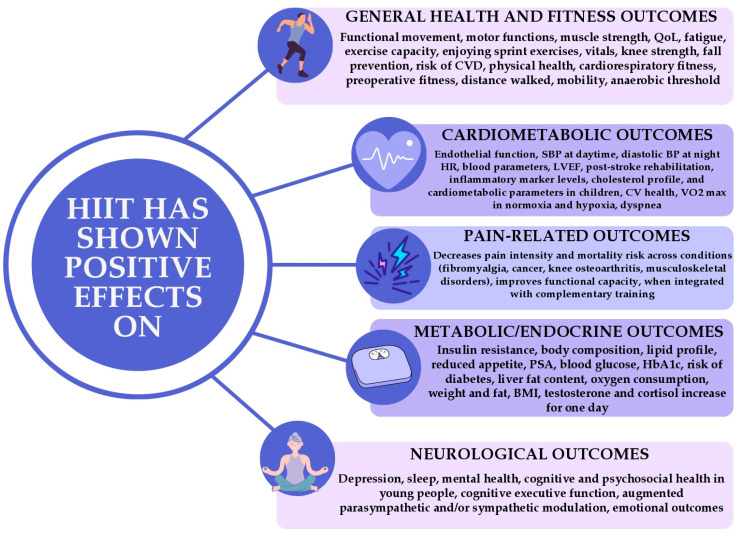
Health benefits of HIIT. Abbreviations: QoL—quality of life, CVD—cardiovascular disease, SBP-Systolic Blood Pressure, BP-Blood Pressure, HR—heart rate, LVEF—Left Ventricular Ejection Fraction, CV—cardiovascular, VO2 max-Maximal Oxygen Consumption, PSA—Prostate-Specific Antigen, HbA1c-Glycated Hemoglobin, BMI-Body Mass Index.

HIIT has been found to have significant physiological benefits (Figure 1), including reducing arterial peripheral resistance, SBP, DBP, blood glucose (BG), improvement in pancreatic β-cell function, and insulin sensitivity/secretion, increase in GLUT-4 expression in skeletal muscles waist circumference (WC), and excess post-exercise oxygen consumption (EPOC). HIIT induces continuous shear stress that can activate potassium channels in endothelial cells and promote greater activity of eNOS, ultimately leading to vasodilation [7,8,9,10,11,12,13,14,15,16,17,18,19,20,21,22,23,24]. Numerous studies showed that loss of lean muscle mass, excessive body fat, and low cardio-respiratory physical state all augment the risk of illness and death. There is a substantial amount of evidence from numerous RCTs showing that HIIT can significantly improve outcomes in patients with cardiac, pulmonary, and metabolic diseases. There is no clear evidence that a short course of HIIT is better than MICT or no exercise for decreasing body fat and increasing muscle mass. However, even a low dose of HIIT was found superior over no exercise and more beneficial than MICT in enhancing cardiorespiratory fitness. It is worth noting that the practicality and effects of performing low-volume HIIT on a larger scale, outside of a lab setting, still need to be discovered [7].

#### 3.2.1. HIIT Exercise-Induced Physiological Potential to Influence Metabolic Syndrome

Supervised low-volume HIIT significantly improved pancreatic β-cell function, which is adjusted for insulin sensitivity and can predict the development of type 2 diabetes. The mechanisms behind the improved pancreatic β-cell function could be multifaceted and may involve anti-inflammatory cytokines secreted by adipocytes and myocytes. It remains to be determined whether long-term exercise has an insulinogenic effect in a T2D population [8,9].

HIIT is potentially capable of improving metabolic syndrome [8]. HIIT positively impacted various health markers such as blood pressure, blood glucose, and waist circumference in people with metabolic syndrome. However, there was only a slight increase in HDL cholesterol levels and no significant effect on triglyceride levels. The decrease in blood glucose levels after HIIT may be due to increased blood flow, skeletal muscle mass, and insulin receptors, which lead to enhanced glucose disposal in the muscles. HIIT can increase GLUT-4 expression in skeletal muscles, a protein involved in glucose transport [7].

#### 3.2.2. Effects of HIIT on Blood Pressure and Vascular Function

The clinical impact of HIIT in reducing SBP and DBP (by around 4 mmHg) is significant as even small decreases of 2 mmHg can lower the incidence of various cardiovascular outcomes such as CAD, MI, stroke, and death. The pathways behind these blood pressure declines have yet to be fully understood. However, potential explanations include improved baroreflex control of sympathetic nerve activity, decreased circulation of norepinephrine (postexercise), decreased total peripheral resistance, and changes in vasodilator and vasoconstrictor factors, all triggered by exercise. Although the extent to which intensity affects the reduction in systolic and diastolic blood pressure is not entirely clear, HIIT appears to be more effective at reducing these measures compared to other types of exercise sessions [8,9].

As the exercise becomes more intense, blood flow and shear stress also increase. In contrast, sedentary individuals who experience chronic low-shear stress due to inactivity are more susceptible to elevations in biomarkers related to vascular dysfunction. These include pro-inflammatory factors, reduced antioxidant expression, oxidative stress, and cell adhesion molecules. HIIT may induce continuous shear stress that can activate potassium channels in endothelial cells and promote greater activity of endothelial nitric oxide synthase (eNOS), ultimately leading to vasodilation. However, studies comparing HIIT and other types of training found no difference in shear rate, suggesting that HIIT may enhance vascular function through other mechanisms beyond increasing shear stress. Yet, only HIIT significantly increased NO bioavailability [10].

Moreover, the researchers speculated that there may be an exercise intensity threshold beyond which nitric oxide (NO) availability could be compromised, as indicated by elevated production of reactive oxygen species (ROS) and decreased circulating antioxidants at higher intensities of physical activity. It is crucial to note that avoiding intensive exercise over the recommended intensity threshold can prevent negative effects on vascular function. The HIIT protocol, which involves short bouts of HIIT with a recovery period between high-intensity intervals, could be a safe and effective way to avoid the adverse effects of excessive HIIT [10].

HIIT was superior to moderate-intensity continuous training MICT in enhancing vascular function. HIIT can improve brachial artery flow-mediated dilation more than MICT, which aligns with previous research showing an association between cardiorespiratory fitness and flow-mediated dilation. HIIT has also been found to enhance cardio-respiratory function more than MICT. The ability of HIIT to mitigate common CVD risk factors, such as insulin resistance, oxidative stress, and inflammation, may explain its superiority in enhancing vascular function. Additionally, HIIT has a more pronounced effect on the cardio-respiratory physical state and CVD-related biomarkers than MICT.

The superiority of HIIT of increasing vascular function over MICT might be explained by its capacity to stimulate enhanced blood flow through the vasculature, providing oxygen to the engaged muscles and promoting greater stress-induced NO bioavailability [10].

#### 3.2.3. HIIT in PCOS

Using only HIIT is a successful method for decreasing BMI in women with PCOS. The research indicates that HIIT enhances certain clinical results in these patients, highlighting exercise’s potential for being an intervention for PCOS not requiring medication [9].

The potential of HIIT to decrease the homeostatic model assessment parameter in women with PCOS may stem from its incorporation of short bursts of intense exercise, such as reaching or surpassing 90% of VO2 max or maximum heart rate. HIIT offers a time-efficient training method that enhances mitochondrial function in muscles, leading to increased insulin sensitivity and biogenesis stimulation [9]. Activation of mitochondria during HIIT results in heightened energy production and improved skeletal muscle oxidation capacity. Furthermore, HIIT programs demonstrate superior antioxidant adaptations compared to sustained moderate-intensity exercise. These findings suggest that HIIT may be more effective in reducing the homeostatic model assessment parameter than moderate-intensity exercise, potentially due to its ability to mitigate oxidative stress, which plays a crucial role in insulin resistance development. Previously, excess adiposity was linked to deteriorating health in women with PCOS, and lifestyle-induced changes in body composition over approximately six months were associated with restored ovulation in obese women with PCOS. This underscores the connection between physical exercise and improved health outcomes in women with PCOS, likely mediated through adiposity reduction. However, this hypothesis may not hold for women with PCOS who maintain a normal BMI [9].

#### 3.2.4. HIIT for Cardiometabolic Correction in Children

HIIT might be an efficient exercise for improving and reducing cardiovascular risks and enhancing overall well-being in the pediatric population. Subsequent research should allow a longer time for follow-up when designing their study to evaluate if the positive benefits achieved through HIIT are sustained over the long term [11].

#### 3.2.5. HIIT After Myocardial Infarction

In post-MI patients, HIIT was more advantageous in augmenting peak VO_2_ compared to control groups without causing any adverse effects. Further analysis revealed that HIIT was more effective than MICT and standard exercise in improving peak VO2. However, there were no significant differences in the effects of HIIT versus MICT or usual physical activity on blood pressure, peak and resting heart rate, left ventricular ejection fraction, left ventricular end-diastolic volume, and QoL [13].

The effectiveness of HIIT in lowering blood pressure in post-MI patients is debated. Some studies argue that HIIT has a superior effect than MICT, while others do not find a significant difference. HIIT lowered heart rate and reduced arrhythmic events, but there was no significant effect on LVF and remodeling in cardiac patients. The evidence suggests that HIIT is safe for cardiac rehabilitation, but the safety concern for post-MI patients requires further evaluation.

Additionally, HIIT and traditional protocols of training showed comparable safety results for post-MI patients undergoing cardiac rehabilitation, without differences in the incidence of cardiovascular complications or injuries provoked by exercise in the HIIT groups compared to the control groups [12].

Although, in middle-aged and older adults, both MICT and interval training demonstrated significant positive changes in cardio-respiratory fitness. However, in comparison to MICT, HIIT and sprint interval training led to greater increases in maximal oxygen uptake (VO_2max_) [13,14].

#### 3.2.6. HIIT in HF

The exercise program for individuals with HF usually involves aerobic exercise, either continuously or in intervals. Research has shown that HIIT can increase aerobic capacity more than continuous exercise in individuals with HF. However, the evidence is insufficient to conclude that HIIT is superior to continuous training [15].

HIIT is a successful technique in treating HF and CAD as it enhances peak VO_2_, with HF patients experiencing a notably greater increase. To maximize the advantages, HF patients should have active recovery intervals at intensities ranging from 40% to 60% of peak VO2. The frequency of training should be 3 d/wk for HF patients and 2 d/wk for CAD patients [16].

#### 3.2.7. Neurological and Psychological Outcomes

The majority of research investigating the relationship between HIIT and sleep has been conducted on adults [17].

HIIT was associated with better sleep outcomes and reduced psychological distress compared to non-active controls, and it was generally considered safe and well-attended across different populations. Overall, these findings provide evidence for the use of HIIT as a means of improving mental health [18].

Engaging in HIIT may enhance the cognitive capacity and psychological well-being of minors. The immediate effects of HIIT were found to be more potent than its long-term effects on cognitive function. Furthermore, HIIT interventions resulted in positive changes in both positive and negative mental states. However, given the scarcity of studies and the considerable variability in their outcomes, further high-quality research is required to validate these results [19].

HIIT and MICT have comparable effects on cardiorespiratory fitness outcomes, but HIIT might offer a slight advantage over MICT for alleviating depression. HIIT shows promising results in improving cardiorespiratory fitness and depression but does not seem significantly affect metabolic factors. Experiments involving physical activity have a crucial role in enhancing physical and mental health indicators for individuals with serious mental illness and should be included in their multidisciplinary management. People with serious mental illness ought to be motivated to select a physical activity program that complies with their abilities and preferences, and HIIT is a viable option that could be beneficial for those willing and able to participate in it [20].

Various methods of HIIT have been found to have positive and relevant short-term effects on academic performance and learning behavior. This suggests that HIIT interventions could be helpful in combating the sedentary lifestyle epidemic and enhancing cognitive abilities in young people [21].

#### 3.2.8. HIIT in Persons with Spinal Cord Injury

PwSCI (persons with spinal cord injury) can improve their cardiorespiratory fitness by engaging in HIIT and continuous resistance training. Almost all types of vigorous exercise interventions have significantly improved cardiorespiratory fitness, suggesting that PwSCI should include such exercises in their usual physical activity routines. However, no significant differences were observed between exercises of high intensity and the standard moderate-intensity ones. Most of the included studies involved few participants with poor fitness levels, indicating the necessity for high-quality studies examining the effect of exercise of varying intensity on physical state. Subsequent RCTs should recruit more participants and ensure recruited PwSCI are physically active [22].

#### 3.2.9. Oncological Outcomes

Due to its intense nature, the feasibility of incorporating HIIT into cancer patients’ clinical pathways should be carefully evaluated. According to the existing SRs and MAs, HIIT was not significantly superior to usual care, although it showed greater effectiveness over programs of moderate intensity. The “Preoperative Exercise to Improve Fitness in Patients Undergoing Complex Surgery for Cancer of the Lung or Oesophagus (PRE-high-intensity interval training)” study protocol aims to verify if HIIT can improve preoperative fitness. The study will analyze participants’ adherence and the effects of HIIT on VO_2_peak, advancing knowledge in this field [23].

Short-term HIIT has demonstrated itself as more effective than usual care (UC) in improving physical fitness and health-related outcomes in oncological patients. However, it is uncertain whether it has a clear advantage over moderate-intensity continuous training. Therefore, using HIIT for cancer patients, especially those with time constraints, during and after treatment may be beneficial [24].

#### 3.2.10. The Impact of HIIT on Pain-Related Disorders

HIIT for Fibromyalgia: Combining HIIT with strength and flexibility exercises, as well as MICT with strength and flexibility exercises, led to significant improvements in fibromyalgia symptoms, pain levels, functional abilities, and overall quality of life when compared to a control group [136].

HIIT for Cancer-Related Fatigue and Pain: Both HIIT and combined HIIT interventions have shown significant efficacy in reducing cancer-related fatigue (CRF) and associated pain. Despite earlier safety concerns, HIIT and combined HIIT programs might be considered a safe, effective, and time-efficient training modality to reduce CRF and pain in cancer patients as well as survivors. [137].

HIIT for Musculoskeletal Pain Conditions: Evidence suggests that HIIT interventions for musculoskeletal disorders can reduce pain intensity and improve VO2 max, but not disability or quality of life. Sub-analyses indicate that HIIT is not superior to other exercise models in alleviating pain. While HIIT can be implemented to improve pain intensity or cardiorespiratory fitness, it is essential to note that changes in pain intensity may not correlate with improvements in quality of life or disability. Pain intensity scores in patients are inversely linked with VO_2_ max, a crucial predictor of cardiovascular health and overall mortality. Individuals with chronic pain and musculoskeletal disorders have elevated risks of cardiovascular and chronic diseases, as well as mortality due to cardiac issues. Improving cardiorespiratory capacity, which HIIT effectively achieves, was shown to decrease mortality risk by up to 16% [37].

In knee osteoarthritis, high-intensity strength training demonstrates similar efficacy in improving knee pain, function, and quality of life compared with low-intensity strength training and standard care with comparable safety [138].

Taken together, these findings suggest that HIIT serves as an effective modality to reduce pain and enhance physical function across a spectrum of pain-related conditions. Although its impact on quality of life remains inconsistent, HIIT may indirectly reduce pain-related morbidity.

### 3.3. Quality Assessment Results

The AMSTAR-2 analysis (Table S1 Supplementary Material) has been performed based on 16 critical and non-critical domains. The distribution of the overall confidence rating is as follows: 5 articles with high confidence, 20 articles with moderate confidence, 27 articles with low confidence, and 82 articles with critically low confidence. The analysis confirms that certain critical domains are systematically unmet: 99 articles did not clearly state protocol registration or did not state it at all (item 2). 86 articles did not clearly justify the exclusion of individual studies (item 7). 95 did not properly assess the risk of bias in individual studies (item 9). 45 did not consider the risk of bias when interpreting the results of the review (item 13). 75 did not properly assess the publication bias (item 15). On the other hand, only 2 articles did not provide appropriate meta-analytical methods, and only 8 articles did not provide adequate literature search (item 4).

### 3.4. Consistency and Discrepancies and Reported Mechanisms

A new table has been created (Table 2) that summarizes the direction of results (effect) for every included study by category. 84 studies belonged to the cardiometabolic category: 64 studies reported positive effects, 17 reported comparable effects, 2 noted “depends on personalized approach,” and 1 study noted “unclear.” These findings suggest a consistently beneficial direction for cardiometabolic outcomes. 13 studies belonged to the neurologic category: 12 studies reported positive effects, and 1 reported comparable effects. Such a prominent positive skew indicates a consistent direction of benefit for neurologic outcomes. 26 studies belonged to the metabolic category: 16 reported positive effects, 7 reported comparable effects, 1 reported inconsistent, 1 did not conclude, and 1 was not applicable. Most findings were positive, but the presence of comparable results indicates some variability in metabolic outcomes. 8 studies belonged to the oncologic category: 5 reported positive effects, 2 comparable, and 1 not significant. These findings suggest partial consistency with occasional discrepancies. 3 studies focused predominantly on pain-related outcomes and 2 reported a comparable effect while 1 reported positive effect. No definitive trend can be inferred.

The tabulation of reported mechanisms (Table 2) revealed a dichotomy: superficial and in-depth reported mechanisms. The cardiometabolic category exhibits the widest range of mechanisms. The general outcomes include “Increased oxygen uptake” and “Peripheral muscle and central cardiorespiratory adaptation”. The more detailed mechanisms consistently converge on three interconnected molecular pathways. The first pathway revolves around Mitochondrial Biogenesis. Mechanisms reported include “Mitochondrial adaptations,” “increases in citrate synthase maximal activity,” and the activation of adenosine monophosphate-activated protein kinase (AMPK). AMPK activation boosts proliferator-activated receptor gamma coactivator 1-alpha (PGC-1α) expression, a regulator recognized for its central role in energy metabolism. The molecular cascade manages the cell’s capacity for sustained energy production and oxidative capacity. The second pathway involves endothelial function and Nitric Oxide (NO) bioavailability. An increase in NO concentration in endothelial cells enhances dependency on peripheral vascular compliance and improves the function of endothelial progenitor cells. Such adaptations are crucial for reducing peripheral resistance and improving arterial flexibility. Thirdly, the central cardiorespiratory changes are also reported. This includes increased stroke volume and ejection fraction induced by enhanced left ventricular systolic function, alongside adaptations in baroreflex-mediated modulation of the sinoatrial node. Metabolic category mechanisms are more molecularly defined. The key specific mechanism is the enhancement of insulin sensitivity and glucose uptake. This is achieved through the increased translocation of Glucose Transporter Type 4 (GLUT-4) to the plasma membrane. GLUT-4 facilitates the diffusion of plasma glucose into muscle tissue and adipose cells. Moreover, HIIT promotes the activity of glycolytic and oxidative enzymes, reduces liver fat, and improves postprandial glucose levels. Likewise, reported mechanisms cite the promotion of glycolipid metabolism, increased fat oxidation, and significant lipolysis driven by increased catecholamine production during exercise. Additionally, HIIT influences hormonal regulation by activating the Hypothalamic–pituitary–adrenal axis, increasing testosterone/cortisol immediately post-session, and chronically modulating appetite by decreasing acylated ghrelin, which may suppress hunger. The neurologic category depicts strong molecular and psychosocial consistency. Most studies report activation of PGC-1α–BDNF pathways, linking metabolic stress (e.g., ↑ H_2_O_2_, TNF-α) to enhanced neurogenesis and cognitive performance. Importantly, psychosocial mechanisms, improvements in self-esteem, mood, and motivation, promote adherence and create a positive feedback loop, where emotional gains sustain physiological adaptation. In oncologic and pain-related categories, mechanisms overlap with cardiometabolic responses, emphasizing mitochondrial biogenesis, angiogenesis, and AMPK-PGC1α activation. Most studies highlight improved oxygen uptake and functional capacity. Targeted molecular effects, such as reduced IL-6, indicate additional anti-inflammatory benefits. A major limitation running across all categories is the tendency for studies to report theoretical or correlational mechanisms.

Studies investigating optimal training duration reported that the maximum benefits are generally between 6 and 12 weeks. Exceeding 6 weeks is necessary to yield significant benefits, particularly for cardiorespiratory fitness and fat loss. Such a window corresponds to the biological timeframe required for structural adaptations, such as mitochondrial biogenesis and vascular remodeling. Regarding frequency, the most commonly cited effective range is 2 to 3 sessions per week. A notable finding indicates that increasing training frequency beyond this minimum may not yield superior molecular outcomes. This suggests that the physiological system may reach saturation at a certain frequency. The high intensity of HIIT drives its unique molecular signature through the recruitment of specific muscle fibers and high metabolic turnover. This includes high speeds to promote the recruitment of type II fast-twitch muscle fibers crucial for the rapid, short-duration power output. Protocols often specify precise high-intensity intervals, such as 4 min at high intensity. The use of adjuncts, such as partial Blood Flow Restriction occlusion and Hypoxic Stimuli, demonstrates an attempt to heighten the acute metabolic stress and subsequent molecular signaling, thereby maximizing the efficacy of the training session.

## 4. Discussion

The current evidence shows that HIIT is beneficial in multiple disorders such as cardiovascular, respiratory, metabolic, neurologic, and oncologic ones. Dozens of studies have confirmed the positive effects of HIIT on the effective management of these diseases. The evidence synthesis revealed a generally uniform benefit of HIIT or similar exercise on cardiometabolic and neurologic outcomes. In the cardiometabolic category of studies, almost all report improvements (VO_2max_, insulin sensitivity, blood pressure). Likewise, in neurologic studies, almost all report positive cognitive or neural benefits. The metabolic and oncologic categories show more mixed patterns, and a few oncologic studies found non-significant changes. This suggests consistency within certain domains (cardiac, fitness, cognition) but greater heterogeneity in metabolic and cancer-related outcomes. The skew toward positive results in nearly every category is notable. Thus, there is concern that negative or null results might be underreported. Such a pattern suggests at least some bias toward publishing favorable results. However, where outcomes were reported as “Comparable” (no difference between HIIT and moderate exercise), this could reflect genuine null findings or underpowered studies. A very high proportion of positive outcomes suggests possible publication bias or positive-reporting bias. This may reflect that many included sources were reviews or guidelines summarizing existing positive trials.

HIIT might not be suitable for some patient populations, including beginners, due to its demanding nature and the necessity for proper warm-up, execution, and cooldown techniques. Novices may lack the necessary form and fitness level for high-intensity workouts, increasing their risk of injury. Individuals with heart conditions or other health issues exacerbated by vigorous exertion should avoid HIIT unless authorized by a medical professional. HIIT poses a higher risk of injury due to its rapid pace and complex movements. Quick execution and poor form increase the likelihood of muscle strains. Moreover, HIIT can lead to overuse injuries and joint strains due to the significant stress it places on the body. Adequate rest between sets and sessions is crucial for injury prevention.

While short-term HIIT has shown superiority over usual care in improving physical fitness and health-related outcomes, its distinct advantage over moderate-intensity continuous training remains uncertain. Therefore, using HIIT for cancer patients who can tolerate this intensity, especially those with time constraints, during and after treatment, may be beneficial. Further studies are encouraged to investigate the effectiveness of HIIT in improving fat-muscle-bone proportions, self-reported effects of the intervention, and serum indicators throughout the intervention and the follow-up period [24].

Additionally, another study revealed that short-term and long-term HIIT interventions yielded favorable outcomes on cardiometabolic health indicators [2]. This further highlights the potential of HIIT as a versatile intervention for improving various physiological functions, including body composition, VO_2max_, endothelial function, muscle strength, functional movement and motor functions, exercise capacity, systolic and diastolic blood pressure, resting heart rate, pain, QoL, depression, LVEF, glycemic control and insulin resistance, lipid profile and blood glucose, post-stroke rehabilitation, fall prevention, liver fat content, preoperative fitness, cognitive, psychological and mental health, executive functions and quality of sleep.

The reliance on systemic outcomes and the theoretical nature of many molecular pathways underscores a critical evidence gap. The existence of numerous “Comparable” or “Inconsistent” results in the analysis, despite strong theoretical mechanistic potential, highlights the non-uniform translation of molecular signals into functional superiority. This suggests that the effectiveness of HIIT is highly context-dependent and variable, demonstrating that the underlying molecular potential does not translate uniformly without robust, individualized protocols. While the specific biological action of HIIT appears to center on superior peripheral vascular remodeling (evidenced by the (DBP) effect) and potent molecular signaling cascades (AMPK/PGC-1alpha), the most critical factor dictating whether an individual successfully activates and sustains these pathways is long-term adherence.

### 4.1. Implications for Future Research

Additional studies are required in various applications of HIIT in clinical medicine including metabolic, cardiovascular, pulmonary, cancer, etc.

Although the metabolic effects of HIIT on vascular function have been extensively studied, further investigation is required to understand the mechanism through which HIIT induces metabolic changes and to identify the lower-end thresholds of frequency and intensity, and what intervals of HIIT exercise are needed to achieve superior effectiveness [7].

Future research should also explore the impact of HIIT on other physical fitness areas, such as sprinting ability, running performance, and countermovement jumping [24].

Given the numerous health benefits of HIIT, it of utmost importance for future studies to delve into the effects of HIIT on the overall health and sleep quality of children and adolescents. This research area holds significant implications for their well-being and development. It is also crucial to explore different forms of HIIT and compare HIIT with other types of exercise in future studies [17].

Physical education teachers, professionals in sports sciences, educators, and researchers should take this into account in the future work. Additional studies spanning over a longer time are necessary [21].

Additional studies are required to assess the safety and effectiveness of HIIT in cardiac rehabilitation, with a larger number of participants, increased duration of follow-up, stratification of patients for cardiac rehabilitation risk, and the consideration of the effect of cardiac medications on outcomes [12].

More studies are needed to assess the effects of HIIT on outcomes in spinal cord injury patients.

Most of the included studies involved a low number of participants with poor fitness levels, indicating the necessity for high-quality studies that examine the effect of exercise of varying intensity on physical state. Subsequent RCTs should recruit more participants and ensure that the recruited PwSCI are active [22]. Future research is also needed to evaluate the impact of HIIT on outcomes in cancer patients. Specifically, future studies should establish the level of intensity at which significant changes can be achieved close to the operation day and to analyze the acceptability and feasibility of HIIT programs [23].

### 4.2. Limitations

Although HIIT has existed for decades, the evidence is still growing. Since we focused on SRs rather than stand-alone experimental studies, we might not have captured recently published primary research articles. HIIT included heterogeneous and multimodal types of HIIT protocols and other exercises, which can add heterogeneity to our conclusions. Moreover, there was heterogeneity in the diseases and outcomes studied.

AMSTAR-2 analysis revealed that most of the studies lacked critical items 2, 7, 9, 13, 15. This raises concern about selective reporting and post hoc decision bias (due to item 2), study selection bias (due to item 7), confidence in pooled evidence quality (due to item 9 and 13), and selective publication (due to item 15).

Protocol variability is another major challenge. Studies vary significantly in interval duration, intensity, recovery time, and overall intervention duration, making cross-study comparisons difficult. Inconsistent reporting of workload and compliance also reduces the reproducibility of results and the interpretation of dose–response relationships. Finally, short follow-up periods (often ≤ 12 weeks) make it difficult to assess long-term sustainability, relapse rates, and physiological maintenance. Few studies assess whether improvements are maintained after the intervention period, leaving the duration of HIIT-induced adaptations uncertain.

These limitations should be considered when conducting future research and implementing this evidence into practice.

## 5. Conclusions

High-intensity interval training has been reported to improve numerous physiological functions and outcomes, including improved body composition, VO_2max_, endothelial function, muscle strength, functional movement and motor functions, exercise capacity, systolic and diastolic blood pressure, resting heart rate, pain, QoL, depression, LVEF, glycemic control and insulin resistance, lipid profile and blood glucose, post-stroke rehabilitation, fall prevention, liver fat content, preoperative fitness, cognitive, psychological and mental health, executive functions, and quality of sleep.

High-intensity interval training was either superior or at least non-inferior compared to moderate-intensity continuous training in improving health outcomes in patients with diabetes mellitus type 1 and 2, obesity, coronary artery disease, myocardial infarction, post-MI syndrome, heart transplantation, cancer, chronic heart failure, coronary artery bypass surgery, arrhythmias, stroke, atherosclerosis, prehypertension, hypertension, asthma, chronic obstructive pulmonary disease, Parkinson’s disease, musculoskeletal disorders, metabolic syndrome, polycystic ovary syndrome, schizophrenia, nonalcoholic fatty liver disease, spinal cord injury, Alzheimer’s disease, anxiety, eating and stress disorders, chronic pain syndromes, as well as several cancers including lung, breast, bladder, rectal, liver, rectal, and testicular.

Nevertheless, the included reviews reported heterogeneity in populations and protocols. Future research should focus on standardizing HIIT protocols, clarifying the minimum effective dose, and evaluating safety and feasibility in vulnerable groups. Additionally, large-scale, long-term randomized trials are needed to assess sustained effects on health outcomes. Practitioners and educators are encouraged to incorporate evidence-based HIIT programs with appropriate supervision and individualized intensity adjustments.

In summary, high-intensity interval training is a powerful stimulus triggering numerous physiological adaptations that can have a significant impact on outcomes in patients with various cardiovascular, respiratory, metabolic, neurologic, oncologic, pain-related conditions.

## Figures and Tables

**Table 1:** Study characteristics.

Author, Citation	Study Design	Study Goals	N of Patients Included in the Analysis	Total Number of Studies Included in the Meta-Analysis	Diagnosis	Comorbidities	Study Conclusions
[117]	SR	Cardiac autonomic responses	193	6	Healthy, CAD, CHD, MetS	-	Augmented parasympathetic and/or sympathetic modulation
[106]	SR + MA	VO_2max_	295	6	COPD	-	HIIT is similar in improving VO_2max_ in comparison with traditional exercises
[84]	SR	Executive function	720	24	Healthy	-	HIIT improves executive function
[21]	SR	Cognitive and psychological outcomes	652	8	Healthy teenagers	-	HIIT improves cognitive and psychological health in young people
[40]	SR	Body composition, muscle strength, physical function	615	8	Healthy	-	HIIT improves body composition and muscle strength
[115]	MA	Anthropometric outcomes	1222	48	Overweight, obese, healthy	-	Comparable results between HIIT and MIIT
[54]	SR + MA	QoL, VO_2max_	375	9	Stroke	-	HIIT enhanced post-stroke rehabilitation and VO_2max_
[15]	SR + MA	VO_2max_, QoL	129	7 in qualitative review, 5 in meta-analysis	Heart failure (HF)	-	Comparable advantages of HIIT inVO_2_ peak, QoL, LVEF
[45]	SR + MA	SV, blood volume, hematocrit, VO_2max_	946	45	Overweight, obesity, COPD, schizophrenia, HF, CAD, MetS, T2DM	-	HIIT improves VO_2max_, and consequently blood parameters
[16]	SR + MA	VO_2max_	404	19	CAD, HF	-	More effective results of HIT in HF than CAD patients
[2]	SR + MA	Cardio- and metabolic outcomes	2164	65	Overweight, obese, healthy patients	-	Improvement of short-term and long-term HIIT on VO_2max_ in healthy adults
[81]	SR + MA	BP, insulin resistance, intrahepatic fat	3033	54	Overweight or obesity	HTN, T2DM, Metabolic syndrome, NAFLD, Dyslipidaemia, NASH	HIIT improves cardiometabolic outcomes
[52]	SR + MA	HR, VO_2max_, glucose, BP, insulin, insulin resistance	707	11	Healthy children	-	HIIT improved exercise capacity and glucose levels
[101]	SR + MA	VO_2max_	480	15	Health and unhealthy	-	HIIT improves VO_2max_
[61]	SR + MA	VO_2max_	438	18	Healthy	-	Sprint interval exercise improves VO_2max_
[66]	SR + MA	VO_2max,_ QoL, exercise capacity, cardiac parameters	515	11	HF	-	HIIT improves VO_2max_ and exercise capacity
[108]	SR + MA	Efficacy, safety	228	11 articles describing 7 studies	MS	-	Safety and efficacy of HIIT
[109]	MA	CRF	563	17	Healthy minors aged < 18	-	Favorable outcome for CRF in HIIT than MICT
[67]	SR + MA	VO_2max_, BP, body composition, glucose, insulin, insulin resistance, lipid profile	325	12	Children with overweight, obesity	-	HIIT improves VO_2max,_ and SBP
[49]	SR + MA	Lipid profile, blood glucose, insulin, insulin resistance	538	18	Children with MetS	Obesity, overweight, asthma, NAFLD	HIIT improves lipid profile and blood glucose
[62]	SR + MA	BP	266	10	HTN	Parkinson, urology, HF	HIIT decreases BP
[33]	SR + MA	Body composition, HR, BP, CRP	380	8	MSK disorders	-	HIIT affected only heart rate
[39]	SR + MA	VO_2max_, fatigue, inflammatory markers	215	6	Prostate cancer	-	HIIT improves VO_2max,_ fatigue, PSA, but not inflammatory markers
[42]	SR	VO_2max,_ blood lactate, creatine kinase	244	18	Healthy	-	HIIT improves exercise capacity
[88]	SR + MA	VO_2max_, heart rate	212	5	Heart transplantation	-	HIIT improves VO_2max_ and heart rate
[120]	SR + MA	BP, VO_2max_	143	9 in qualitative review, meta-analysis: 9 for VO_2max_, 7 for BP	Pre-HTN, HTN	CHF, CHD, MetS, abdominal obesity, pre-T2DM	No difference in BP at rest, improved VO_2max_
[37]	SR + MA	Pain, VO_2max_, QoL	530	13	MSK disorders	-	HIIT decreases pain, improves VO_2max_, but not QoL
[113]	SR	BG	325 (drop-out 182)	5	T2DM	-	No sufficient evidence
[125]	SR + MA	Functional capacity and cardiometabolic outcomes	184	7 in qualitative review, 5 in meta-analysis	Pre- and T2DM	-	More favorable functional capacity of HIIT but comparable cardiometabolic outcomes of HIIT and MICT
[48]	SR	Glucose, inflammatory markers, lipid profile	168	14	T2DM, T1DM	-	HIIT improves glycemic control
[32]	SR + MA	Exercise capacity, BP, VO_2max_	569	9	HTN	-	HIIT is superior to moderate intensity exercise in improving VO_2max_
[112]	SR	Body composition, CRF	136	6	Overweight	-	Favorable effect of HIIT on body composition
[79]	SR + MA	Testosterone and cortisol	890	60	Healthy	-	Testosterone and cortisol increase immediately and return to baseline in one day
[93]	SR + MA	Body composition	959	38	Females	Overweight, obesity, T2DM, PCOS, dislipidemia, rheumatic disease, metabolic syndrome	HIIT helps reducing weight and fat
[63]	SR + MA	BP, HR	1583	38	HTN	-	HIIT decreases BP worse than isometric exercise training, but better reduces HR
[68]	SR	Fall risk factors, physical activity, QoL	328	11	Healthy	-	HIIT is safe and effective in fall prevention
[134]	MA	VO_2_ peak	229	6	CAD (CABG, AP, MI, PCI)	-	More favorable outcome for VO_2max_ and anaerobic threshold in HIIT
[73]	SR	Anthropometric and CVS parameters, lung function, cardiorespiratory fitness, asthma symptoms and control, QoL	841	7	Asthma	-	HIIT improved FEV1 and oxygen consumption
[75]	SR + MA	Pain-free walking distance and oxygen consumption	1132	19	Lower extremity PAD	-	HIIT was less effective in walking distance than light-to-moderate PA, but more effective in maximal oxygen consumption
[70]	SR + MA	Pulmonary function, dyspnea, QoL, adverse events, VO_2max_	689	12	COPD	-	HIIT improves pulmonary function, QoL, dyspnea, VO_2max_
[130]	SR + MA	Cardiometabolic outcomes and VO_2max_	274	9	Overweight and obese children < 18	-	More favorable outcome in BP and VO_2max_ for HIIT
[36]	SR + MA	Pulmonary function, VO_2max_, muscle strength	399	12	CF	-	HIIT improves VO_2max,_ muscle strength, but not lung function
[127]	SR + MA	VO_2max_, QoL	609	12	CAD	-	Improved VO_2max_ for HIIT, but no difference in QoL
[122]	SR + MA	VO_2max_	411	13	HFREF	-	Superiority of HIIT over MICT in VO_2max_
[41]	SR + MA	Severity of depression and anxiety	515	12	CAD, angina, arrhythmias, HF, HTN, stroke, MI, atherosclerosis, CMP, Parkinson	-	HIIT improves depression, but not anxiety
[51]	SR + MA	Body composition, VO_2max_, glucose, insulin	230	9	Overweight, obesity	-	HIIT and fasting improve glucose
[43]	SR + MA	VO_2max_, exercise capacity	846	55	Healthy	-	HIIT improves exercise capacity
[124]	SR + MA	CRF, AE	953	17	CAD (MI, PCI, CABG, PTCA)	-	Improved CRF in HIIT group. No difference in AE
[28]	SR + MA	Safety, VO_2max_	117	11	Parkinson	-	HIIT is safe, improves VO_2max_ and motor functions
[135]	SR + MA	VO_2_ peak, LVEF	168	7	HFREF	-	Higher effectiveness of HIIT than MICT on VO_2max_ improvement
[72]	SR + MA	VO_2max_	305	8	Lung cancer	-	Favorable effects of HIIT on oxygen consumption
[91]	SR	Executive function, heart rate, VO_2max_	1223	23	Healthy children and adults	-	HIIT improves cognitive executive functions
[74]	SR + MA	Weight, BMI, fat percentage, oxygen consumption	129	10	All adults	Diabetes, overweight, obese	Diet and HIIT reduce weight and fat
[53]	SR + MA	Exercise enjoyment	675	25	Healthy	-	Participants enjoyed sprint exercises comparable to HIIT
[29]	SR + MA	Appetite	169	13	Healthy	-	Both HIIT and moderate exercise reduced appetite
[131]	MA	T2DM-related outcomes, weight, CRF	2035	50 (36 controlled, 14 one-group)	Healthy, sedentary, overweight, obese	T2DM, MetS, HF, CAD, MI, angina, schizophrenia, cancer	Favorable effects of HIIT on insulin resistance, fasting glucose and HbA1c levels, body weight, and CRF
[99]	SR	VO_2max_	259	15	HF, COPD, T2DM, CAD, cancer	-	HIIT can improve VO_2max_ similar to moderate-intensity exercise
[94]	SR + MA	Inflammatory markers	841	29	Overweight, obesity, T2DM, PCOS, metabolic syndrome, NAFLD	-	HIIT decreases inflammatory markers
[83]	SR + MA	Liver fat percentage	333	10	Overweight or obesity	CAD, NAFLD, T2DM	HIIT improves liver fat content
[34]	SR + MA	Vascular function	1437	36	Overweight, obesity, MetS, T2DM, T1DM, PCOS, HTN, HF, CAD, MI, heart transplant, ToF, cancer	-	HIIT improves vascular function
[50]	SR + MA	Blood glucose, insulin	467	30	MetS	Overweight, obesity, T2DM	HIIT improves insulin and glucose responses
[56]	SR + MA	Glucose, insulin	870	25	T2DM, HTN, obesity, NAFLD, overweight	-	HIIT improves glucose and insulin levels
[20]	SR + MA	Safety, VO_2max_, body composition, psychological health, QoL	366	9	Severe mental illness	-	HIIT shows adherence, is safe, decreases depression, improves VO_2max_
[57]	SR + MA	VO_2max,_ BP, body composition, knee strength, HR, lipid profile	476	13	Healthy	-	Aquatic HIIT improves body composition, lipid profile, vitals, knee strength, and VO_2max_
[19]	SR + MA	Cognitive and mental health	2092	22	Children	-	HIIT can improve cognitive and mental health
[100]	SR + MA	Blood pressure, VO_2max_	269	15	HTN	HF, obesity, overweight, CAD, metabolic syndrome	HIIT decreases blood pressure. It is superior to moderate intensity exercise in improving VO_2max_
[64]	SR	Body composition, inflammatory markers	258	7	T2DM	-	HIIT decreases inflammatory marker levels
[65]	SR + MA	VO_2max_, lipid profile, QoL, cardiac parameters	465	8	HF, MI, ToF	-	HIIT and moderate intensity exercises improve VO_2max_
[69]	SR + MA	Lipid profile, body composition, insulin resistance, VO_2max,_	704	19	Obese children	-	Aerobic exercises reduce the risk of CVD, mixed exercises reduces the risk of diabetes
[30]	SR + MA	BP, HR, VO_2max_	442	13	HTN	-	HIIT better decreases SBP at daytime than moderate exercise
[132]	MA	VO_2max_ and CV outcomes	472	10	CAD	-	Improved mean VO_2max_ in HIIT group
[119]	SR + MA	T2DM control, CRF	345	13	T2DM	-	Superiority of HIIT over MICT or no training on body composition, VO_2_peak, and HbA1c level
[98]	SR + MA	VO_2max_, body composition, blood pressure, lipid profile, blood glucose	309	9	Childhood obesity	-	HIIT improves VO_2max_, body composition, and blood pressure
[105]	SR + MA	Body composition, cardiopulmonary parameters	548	10	T2DM	HTN, obesity, renal diseases, cardiovascular diseases	HIIT improves body composition and cardiopulmonary outcomes
[59]	SR + Network MA	BP, BMI, HR	846	12	HTN	-	Moderate intensity exercise lowers BP better than HIIT. HIIT better improves exercise capacity
[126]	MA	Body adiposity	617	39	Healthy, overweight, obese, sedentary adults,	Pre- and T2DM, MetS, PMW, NAFLD, PCOS, rheumatic disease	Reduction in HIIT of visceral, abdominal, and total fat
[90]	SR + MA	VO_2max_	1417	29	MI	-	HIIT improves VO_2max_
[97]	SR + MA	VO_2max_	1201	18	Overweight, obesity teenagers	-	HIIT improves VO_2max_
[103]	Meta-review	VO_2max_, body composition, blood glucose, blood pressure, inflammatory markers, exercise capacity, cognitive and mental health, QoL, safety, adherence	19566	33	DM, metabolic syndrome, HF, CAD, COPD	-	HIIT is beneficial for physical and mental health, its adherence is high, and it is safe
[104]	SR + MA	VO_2max_, mental health, body composition, inflammatory markers, QoL, adverse events	360	12	Mental illness: anxiety, eating, stress disorders	-	HIIT improves physical and mental health
[17]	SR + MA	Effects of HIIT on psychological and physical illness, including sleep	2901	53	General population	Cardiometabolic disorders COPD, Cancer, stroke, Crohn’s disease, Cutaneous systemic sclerosis, and liver resection.	Beneficial effects of HIIT on physical and mental health
[47]	SR + MA	VO_2max_, glucose, weight, glycemic control, insulin, insulin resistance	708	19	T2DM	Metabolic syndrome, obesity, NAFLD	HIIT improves glycemic control and insulin resistance
[31]	SR + MA	Blood glucose	155	15	T1DM	-	Inconsistent effects on blood glucose
[78]	SR + MA	Body composition, lipid profile, blood glucose	422	9	Overweight or obesity, HTN, dyslipidemia, hyperglycemia, insulin resistance	-	HIIT improves body composition, lab analyses, and exercise capacity
[133]	SR + MA	VO_2max_	723	28	Healthy	-	Higher improvement in VO_2max_ in after HIIT than endurance training
[17]	SR + MA	Primary: sleep quality Secondary: anxiety, depression and health-related QoL	755	21	All adults	RA, CKD, testicular cancer, prostate cancer, overweight, obesity, sleep apnea, depression, insomnia, Parkinson, axial spinal arthritis, drug use disorders	HIIT improves sleep
[85]	SR + network MA	Body composition, fat percentage	4774	32	Obesity	-	Aerobic with resistance training can improve body composition
[24]	SR + MA	Physical well-being and health outcomes	448	12	Cancer	Various cancer types	Comparably favorable effect of HIIT and MIE; superiority of HIIT over UC for VO_2max_
[121]	SR + MA	Emotional outcomes	156	8	Active, sedentary	-	Favorable emotional outcomes of HIIT
[89]	SR + MA	Safety, VO_2max_	896	8	Cancer: lung, breast, bladder, rectal, liver	-	HIIT is beneficial and safe
[25]	SR + MA	Fasting glucose, glycemic control, insulin resistance, body composition, lipid profile, BP, VO_2max_	69	5	T2DM	-	HIIT improves glycemic control, insulin resistance, body composition, VO_2max_, and lipid profile
[123]	SR + MA	VO_2_peak, hemodynamic outcomes	118	3	Heart transplant recipients	-	Increased peak HR and VO_2max_ following 8–12 week HIIT
[92]							
[22]	SR + MA	Peak VO_2max_	145	16	spinal cord injury	-	HIIT is beneficial for CVS health, but not superior to other exercises
[80]	SR + MA	Cardiorespiratory parameters	523	21	T2DM	-	HIIT improves cardiorespiratory parameters
[13]	SR + MA	VO_2max_	429	14	Healthy	-	HIIT improves fitness
[107]	SR + MA	Intermittent claudication, VO_2max_	350	9 articles describing 8 studies	Peripheral arterial disease	-	Improvement in distance walked and VO_2max_
[12]	SR + MA	QoL, AEs, vitals, peak VO_2max_, LVEF, LVEDV	387	8	Post-MI	-	HIIT is safe and improves exercise capacity
[10]	SR + MA	Vascular outcomes	182	7	HF, MetS, HTN, T2DM, PMW, obese	-	Enhanced function of BAVF following 12 week or longer HIIT
[14]	SR + MA	Body fat, VO_2max_	784	26	Overweight or obesity	-	HIIT was less effective than moderate exercise in increasing VO_2max_
[71]	SR + MA	Liver fat	745	19	T2DM, NAFLD, obesity, liver steatosis	-	HIIT improves liver fat content similar to moderate intensity exercise
[26]	SR + MA	Endothelial function	208	8	Overweight, obesity	-	HIIT improves endothelial function
[9]	SR + MA	Insulin resistance, BMI	423	7	PCOS	-	HIIT improve insulin resistance and BMI
[38]	SR	QoL, VO_2max_	379	5	CABG patients	CAD, MI	HIIT improves QoL and VO_2max_
[44]	SR + MA	Body composition, VO_2max_, lipid profile, blood glucose	657	22	Healthy	Obesity, sarcopenia	HIIT improves body composition, but is less effective in improving VO_2max_ in comparison with traditional exercise
[8]	SR + MA	Body composition parameters, lipid profile, fasting glucose, blood pressure	414	10	Metabolic syndrome	-	HIIT improves blood pressure, blood glucose levels, and body composition
[23]	SR + MA	Peak VO_2max_	384	5	oncological resections	-	HIIT improves preop fitness
[11]	SR + MA	Changes in BMI, fat percentage, cardiometabolic risk factors, heart rate, oxygen consumption	512	11	Children	Overweight, obesity	HIIT improves cholesterol profile and cardiometabolic parameters in children
[27]	SR + MA	Functional movement	851	18	HTN, obesity, Alzheimer, COPD, CAD, HF, CAD	-	HIIT improves functional movement
[114]	MA	CVD outcomes	620	22	Overweight/obese	-	Improved body composition, TC, VO_2max_ in HIIT group
[7]	SR + MA	Body composition, VO_2max_	1422	47	Overweight, obesity	T2DM, Down syndrome, MetS, NAFLD, cancer	Improvement in VO_2max_, but not body composition
[86]	SR	VO_2max_	639	12	Breast cancer	-	HIIT improves cardiorespiratory fitness
[118]	SR + MA	LVEF	1078	18	HFREF	-	Superiority of 2- 3-month HIIT on improving LVEF
[95]	SR + MA	VO_2max_, safety	516	12	Cancer: liver, lung, rectal, bladder, breast, testicular	-	HIIT improves VO_2max_
[76]	SR + MA	Peak VO_2max_	543	19	Overweight, obesity	NAFLD, T2DM	HIIT improves cardiorespiratory fitness
[46]	SR + MA	VO_2max_, LVEF	664	15	CAD, HF	-	HIIT improves VO_2max_ and LVEF
[111]	SR + MA	Central arterial stiffness, 24 h BP	491	16	Any health status	-	Reduction in diastolic BP at night in HIIT versus MICT
[35]	SR + MA	VO_2max_	194	9	Healthy	-	HIIT improved VO_2max_ in normoxia and hypoxia
[128]	SR + MA	Body composition	424	13	Overweight/obese adults aged 18–45	-	Comparable slight improvement in body composition (but not weight)
[116]	SR	CV outcomes	1117	23	CAD (MI, PCI, CABG)	-	Low risk of CV AE following HIIT
[11]	SR	VO_2max_, mobility	140	6	Stroke	-	Improved VO_2max_ and mobility compared to baseline but not to MICT
[102]	SR + MA	Lipid profile, heart rate, VO_2max_	791	26	HTN, overweight, obesity	-	HIIT and moderate exercise are similar in improving lipid profile. HIIT is superior to moderate exercise in improving HDL
[82]	SR + MA	VO_2max_, body composition, metabolic parameters	1156	29	Older patients	-	HIIT was beneficial at improving fitness
[129]	SR + MA	VO_2max_	736	21	Cardiac patients	-	Improved VO_2_ peak
[60]	SR + MA	Endothelial function	221	9	Healthy	-	Aerobic function improves endothelial function
[58]	SR and MA	VO_2max_	520	10	CHD, HF	-	HIIT improves VO_2max_ and QoL
[55]	SR + MA	VO_2max_	949	22	CAD, HF, MI, heart transplant	-	HIIT improves VO_2max_
[87]	SR + MA	Lipid profile, restenosis, cardiopulmonary parameters	247	6	CAD	-	HIIT improves cardiopulmonary parameters, but not heart rate
[77]	SR + MA	Peak VO_2max_, HR, body composition	488	11	Obesity	-	HIIT reduces fat and BMI
[96]	SR	VO_2max_, body composition, exercise performance	6768	116	Obesity	HF, T2DM	HIIT improves VO_2max_ and body composition
[136]	SR	Physical fitness and functional capacity	834	13	Fibromyalgia	-	Combined training programs are the most effective for patients with fibromyalgia
[137]	SR + MA	VO_2max,_ AE, pain, and QoL	938	12	Cancer-related fatigue	Pain related to cancer	HIIT and combined HIIT can reduce pain and cancer-related fatigue
[138]	SR + MA	VO_2max,_ AE, pain, and QoL	892	10	Knee osteoarthritis	-	HIIT have comparable effects with low-intensity training

Abbreviations: AE—adverse event, BAVF–bronchial artery vascular function, BG—blood glucose, BMI–body mass index, BP—blood pressure, CABG—coronary artery bypass graft, CAD—coronary artery disease, CF—cystic fibrosis, CHD—chronic heart disease, CKD—chronic kidney disease, CMP—cardiomyopathy, COPD—chronic obstructive pulmonary disease, CRF—cardiorespiratory fitness, CRP—C-reactive protein, CV—cardiovascular, CVD—cardiovascular disease, HF—heart failure, HIIT-high-intensity interval training, HR–heart rate, HTN—hypertension, LVEDV-left ventricular end-diastolic volume, LVEF-left ventricular ejection fraction, MA–meta-analysis, MetS—metabolic syndrome, MI—myocardial infarction, MS—multiple sclerosis, MSK—musculoskeletal, N—number, NAFLD—non-alcoholic fatty liver disease, PA–physical activity, PAD–peripheral artery disease, PCOS—polycystic ovary syndrome, PMW—postmenopausal women, QoL–quality of life, RA–rheumatoid arthritis, SBP–systolic blood pressure, SR—systematic review, T1DM–type 1 diabetes mellitus, T2DM—type 2 diabetes mellitus, TC—total cholestretol, ToF–tetralogy of Fallot.

**Table 2:** Mechanisms and direction of effects of included studies.

Included Study	Category	Reported Mechanism and Frequency (If Reported)	Direction
[117]	Cardiometabolic	Parasympathetic and/or sympathetic modulation.	Positive
[106]	Cardiometabolic	Physiological mimicry and lower dynamic hyperinflation.	Comparable
[84]	Neurologic	Raising of H_2_O_2_ and TNF-α activates PGC-1α, which promotes brain-derived neurotrophic factor (BDNF) synthesis. Prefrontal cortex activation. Alterations in lactate and catecholamine levels.	Positive
[21]	Neurologic	Reported best intervals: 4–16 weeks, for 8–30 min/session, psychosocial mechanism: ability to improve self-concept, self-esteem, cognitive ability, and self-perception in youth.	Positive
[40]	Cardiometabolic	Reported no differences between training 1, 2, or 3 days per week. The mechanism involves axonal regeneration for muscle growth promotion.	Comparable
[115]	Metabolic	Decreases body mass.	Comparable
[54]	Cardiometabolic	Increased oxygen uptake.	Positive
[15]	Cardiometabolic	Increased oxygen uptake.	Comparable
[45]	Cardiometabolic	Adaptations in central oxygen transport and/or peripheral oxygen extraction.	Positive
[16]	Cardiometabolic	The maximum benefits are between weeks 6 and 12. The mechanism involves increased oxygen uptake.	Positive
[2]	Cardiometabolic	Duration for at least 12 weeks. Increased baroreflex-mediated modulation of the sinoatrial node.	Positive
[81]	Cardiometabolic	Improvement in insulin sensitivity, glucose uptake, mitochondrial lipid oxidation, and arterial flexibility.	Positive
[52]	Cardiometabolic	At least 5 days duration (for children). Neuromuscular exercises (sit-ups and push-ups) are the most effective for adolescents.	Positive
[101]	Cardiometabolic	Peripheral muscle and central cardiorespiratory adaptation	Positive
[61]	Cardiometabolic	Duration is more than 2 weeks. Increased oxygen uptake.	Positive
[66]	Cardiometabolic	Peripheral mechanisms that lead to ameliorated oxygen utilization by skeletal muscles.	Positive
[108]	Cardiometabolic	Can improve insulin sensitivity and blood pressure.	Positive
[109]	Cardiometabolic	Mitochondrial adaptations, increases in citrate synthase maximal activity, type Ⅱ fiber activation, adenosine monophosphate-activated protein kinase activity, and central adaptation	Positive
[67]	Cardiometabolic	The increased translocation of GLUT-4 to the plasma membrane and the activation of AMP-activated kinase (AMPK). Increased blood flow velocity, elevated nitric oxide (NO) level in endothelial cells, and increased nitric oxide are dependent on peripheral vascular compliance.	Positive
[49]	Metabolic	Glycolipid metabolism promotion.	Positive
[62]	Cardiometabolic	Blood pressure reduction.	Comparable
[33]	Metabolic	Increased oxygen uptake.	Comparable
[39]	Oncologic	Middle- to long-term physiological adaptation, adaptation to high physiological load, increased oxidative enzyme activities, mitochondrial biogenesis, and angiogenesis, activation of AMPK-PGC1α than CAMK-PGC1α (cell stimuli), stimulation of glycogen synthesis	Positive (but is not considered novel)
[42]	Cardiometabolic	Under partial Blood flow restriction occlusion, different haemodynamic and vascular responses are elicited to control the changes in blood flow and alterations in oxygen delivery	Positive
[88]	Cardiometabolic	Recommended 4 min at high intensity. The mechanism involves increased oxygen uptake.	Positive
[120]	Cardiometabolic	Increased oxygen uptake.	Comparable
[37]	Pain-related outcome	Muscular adaptations (mitochondrial biogenesis and increased intramuscular capillarisation), vascular adaptations (increased blood cell volume), and cardiac adaptations (increased cardiac output and contractility).	Comparable
[113]	Metabolic	Increase insulin sensitivity.	Not concluded
[125]	Metabolic	Improves functional capacity.	Comparable
[48]	Metabolic	Increased GLUT-4 in the plasma membrane, improved uptake of muscle glucose, and an increase in the activity of glycolytic and oxidative enzymes.	Positive
[32]	Cardiometabolic	Increased oxygen uptake.	Positive
[112]	Cardiometabolic	The reported duration is 6–24 weeks with 2–3 sessions per week. Increased metabolic and cardiorespiratory stress.	Positive
[79]	Metabolic	Testosterone and cortisol increase immediately after a single HIIT session, then drop below baseline levels, and finally return to baseline values after 24 h. Genomic and non-genomic androgen action. Hypothalamic–pituitary–adrenal axis activation.	Not applicable. The study tested how HIIT increases levels of hormones.
[93]	Metabolic	Increased catecholamine production, leading to significant lipolysis during exercise, followed by higher post-exercise fat oxidation.	Positive
[63]	Cardiometabolic	Decreased resting blood pressure	Positive
[68]	Neurologic	Optimal periods are 12 weeks, 2 sessions a week.	Positive
[134]	Cardiometabolic	Improvement in anaerobic threshold.	Positive
[73]	Cardiometabolic	Increased oxygen uptake.	Positive
[75]	Cardiometabolic	A personalized approach may lead to greater improvements in cardiorespiratory fitness.	Depends on a personalized approach
[70]	Cardiometabolic	Can improve pulmonary function	Positive
[130]	Cardiometabolic	Adaptations in muscles’ mitochondrial enzymes, improved ability to extract and use available oxygen.	Positive
[36]	Cardiometabolic	Improving respiratory muscle function, but not the lung function.	Positive
[127]	Cardiometabolic	Increased oxygen uptake.	Comparable
[122]	Cardiometabolic	Increased oxygen uptake.	Comparable
[41]	Cardiometabolic	Reduces inflammation, enhances neurogenesis via increased BDNF, improves hormonal balance by elevating monoamines and regulating the HPA axis, and decreases oxidative stress by boosting antioxidant defenses.	Positive
[51]	Metabolic	Increased aerobic capacity.	Positive
[43]	Cardiometabolic	Increase in cross-bridge cycling and Ca^2+^ movement, elevation of adenosine monophosphate and activation of adenosine monophosphate kinase, which boosts proliferator-activated receptor gamma coactivator 1-alpha (PGC-1α) expression, leading to enhanced mitochondrial adaptations.	Positive
[124]	Cardiometabolic	Improves cardiorespiratory fitness.	Comparable
[28]	Neurologic	Recommended up to 12 weeks duration. May increase BDNF and cardiorespiratory fitness.	Positive
[135]	Cardiometabolic	Increased oxygen uptake.	Positive
[72]	Oncologic	Improves cardiorespiratory fitness.	Positive
[91]	Neurologic	Improves cerebral oxygenation, arousal, and neuroendocrine responses.	Positive
[74]	Cardiometabolic	Decreases insulin in combination with a ketogenic diet.	Positive
[53]	Cardiometabolic	Psychological changes, motivation.	Comparable
[29]	Metabolic	Can decrease acylated ghrelin and may suppress hunger.	Positive
[131]	Metabolic	Reduction in fasting glucose.	Positive
[99]	Cardiometabolic	Improves cardiorespiratory fitness.	Positive
[94]	Metabolic	Improves circulating TNF-α, leptin and adiponectin	Positive
[83]	Metabolic	Reduces liver fat.	Positive
[34]	Cardiometabolic	Increased NO bioavailability, antioxidant capacity, anti-inflammatory effects, and increased abundance of endothelial progenitor cells.	Positive
[50]	Metabolic	Reduces postprandial glucose.	Positive
[56]	Metabolic	Reduces postprandial glucose.	Comparable
[20]	Neurologic	Improves mood.	Positive
[57]	Cardiometabolic	The hydrostatic pressure in the water created by resistance in movement at high speed promotes muscle action. High speeds promote the recruitment of type II fast-twitch muscle fibers.	Positive
[19]	Neurologic	Brain-derived neurotrophic factor and catecholamines induced by exercise may improve cognitive performance.	Positive
[100]	Cardiometabolic	Increased oxygen uptake.	Positive
[64]	Metabolic	No change in inflammatory biomarkers, reduction in the values of weight and abdominal fat.	Positive
[65]	Cardiometabolic	Improves endothelial progenitor cells function, the structure of coronary arteries, and establishes collateral circulation, thereby increasing blood flow and myocardial support. It can regulate vascular tension, improve arterial compliance, and lower the patient’s blood pressure at rest. It can control mood swings, improve negative emotions, maintain the body’s energy balance, and reduce fat accumulation.	Positive
[69]	Cardiometabolic	It can promote free fatty acids in the blood to enter the cells. Improves the activities of lipoprotein lipase and hepatic lipase in the muscle and liver. Can decrease leptin, tumor necrosis factor-α, and interleukin-6. Can improve the expression of the uncoupling protein-3 mRNA in the skeletal muscle and the catecholamine level to promote the metabolism level of adipose.	Positive
[30]	Cardiometabolic	Promotes nitric oxide production by endothelial cells.	Comparable
[132]	Cardiometabolic	Improves resting heart rate and oxygen uptake.	Positive
[119]	Cardiometabolic	Changes in hemoglobin A1c and 2 h glucose.	Positive
[98]	Cardiometabolic	Favors fat utilization during the recovery period.	Positive
[105]	Cardiometabolic	Changes in hemoglobin A1c and average glucose.	Positive
[59]	Cardiometabolic	Increased oxygen uptake.	Depends on a personalized approach
[126]	Metabolic	Reduces whole body adiposity.	Positive
[90]	Cardiometabolic	Improves cardiometabolic fitness	Positive
[97]	Cardiometabolic	Long-term and short-term HIIT are similarly effective. Improves cardiorespiratory fitness.	Positive
[103]	Neurologic	Improvements in anxiety and depression	Positive
[104]	Neurologic	Improvements in motor skills and mental health outcomes.	Positive
[18]	Neurologic	Moderate improvements in mental well-being.	Positive
[46]	Cardiometabolic	Improvement in glycemic control and insulin resistance.	Positive
[31]	Metabolic	Decreases blood glucose.	Inconsistent
[78]	Cardiometabolic	Not reported.	Positive
[133]	Cardiometabolic	Increased oxygen uptake.	Positive
[17]	Neurologic	Small increase in slow-wave sleep (SWS) and total sleep time (TST).	Positive
[87]	Metabolic	Post-exercise oxy-gen consumption and fat beta-oxidation.	Positive
[24]	Oncologic	Not reported.	Comparable
[121]	Neurologic	Not reported.	Positive
[89]	Oncologic	Increased oxygen uptake.	Positive
[25]	Cardiometabolic	Total cholesterol, high-density lipoprotein, low-density lipoprotein and triglycerides blood lipid metabolism	Positive
[123]	Cardiometabolic	Recommended 8–12 weeks. Improved the cardiocirculatory function, stimulating the sinus node faster.	Positive
[92]	Cardiometabolic	Passive recovery improves performance.	Positive
[22]	Cardiometabolic	Not reported.	Positive
[80]	Cardiometabolic	Not reported.	Positive
[13]	Cardiometabolic	Improves cardiorespiratory fitness.	Positive
[107]	Cardiometabolic	Increased oxygen uptake.	Positive
[12]	Cardiometabolic	Lowers blood pressure and increases oxygen uptake.	Positive
[10]	Cardiometabolic	Effects on oxidative stress, inflammation, and insulin sensitivity.	Positive
[14]	Cardiometabolic	Not reported.	Positive
[71]	Metabolic	Enhances cardiorespiratory fitness, mitochondrial function, and fat metabolism.	Comparable
[26]	Cardiometabolic	Improves endothelial function.	Positive
[9]	Metabolic	Promotes translocation of GLUT-4 receptors inside the cell of the membrane, facilitates the diffusion of plasma glucose into striated muscle tissue and adipocytes without the need for insulin action.	Positive
[38]	Cardiometabolic	Not reported	Comparable
[44]	Cardiometabolic	Improves stretch-shortening cycles that favor recruitment of type 2 muscle fibers and thereby promote muscle hypertrophy	Comparable
[8]	Metabolic	Greater fat oxidation, and changes in appetite and satiety.	Positive
[23]	Oncologic	Not reported.	Not significant
[11]	Cardiometabolic	Improves total cholesterol, low-density lipoprotein cholesterol, and triglycerides levels in children.	Positive
[27]	Neurologic	Adaptations to increased oxygen uptake.	Comparable
[114]	Cardiometabolic	Improves cardiorespiratory fitness and increase in skeletal muscle mitochondrial respiration.	Comparable
[7]	Cardiometabolic	Improves cardiorespiratory fitness.	Comparable
[86]	Oncologic	Increases lower body muscle mass, endothelial function, can reduce interleukin-6 biomarker.	Positive
[118]	Cardiometabolic	Not reported.	Comparable
[95]	Oncologic	Increased oxygen uptake.	Comparable
[76]	Cardiometabolic	Increases stroke volume and ejection fraction of the heart induced by enhanced left ventricular systolic function, increases peroxisome proliferator-activated receptor γ-coactivator-1α (PGC-1α) and glucose transporters following, induces improved mitochondrial function.	Unclear
[46]	Cardiometabolic	Improves mitochondrial function at the molecular level.	Positive
[111]	Cardiometabolic	Decreases blood pressure.	Positive
[35]	Cardiometabolic	Hypoxic stimuli improve HHIT effectiveness.	Positive
[128]	Metabolic	Decreases whole body fat.	Comparable
[116]	Cardiometabolic	Not reported.	Positive
[11]	Cardiometabolic	Not reported.	Positive
[102]	Metabolic	Not reported	Comparable
[82]	Cardiometabolic	Improves cardiorespiratory fitness.	Positive
[129]	Cardiometabolic	Increases oxygen uptake.	Positive
[60]	Cardiometabolic	Recommends 8 weeks. Improves endothelial function.	Positive
[58]	Cardiometabolic	Increases cardiac pumping function and improves cardiopulmonary exchange function.	Positive
[55]	Cardiometabolic	Improves cardiorespiratory fitness.	Positive
[87]	Cardiometabolic	Improves cardiopulmonary function.	Comparable
[77]	Metabolic	Reduces body fat percentage.	Positive
[96]	Metabolic	Increased oxygen uptake.	Positive
[136]	Pain-related outcome	Minimum 14 weeks is recommended. Modulation of the HPA axis and increased serotonin, norepinephrine and endogenous opioid activity, enhancing pain-inhibitory pathways.	Positive
[137]	Oncologic	Not reported	Positive
[138]	Pain-related outcome	Not reported	Comparable

## Data Availability

No new data were created or analyzed in this study. Data sharing is not applicable to this article.

## Supplementary Materials

The following supporting information can be downloaded at https://www.mdpi.com/article/10.3390/jcm14238328/s1, Table S1. AMSTAR-2 analysis of included studies.

## Abbreviations

The following abbreviations are used in this manuscript:

HIIT	high-intensity interval training
VO_2max_	maximal oxygen uptake
HR	heart rate
BP	blood pressure
AE(s)	adverse event(s)
QoL	quality of life
CAD	coronary artery disease
HF	heart failure
MI	myocardial infarction
BMI	body mass index
HTN	hypertension
T2DM	type 2 diabetes mellitus
SBP	systolic blood pressure
DBP	diastolic blood pressure
BG	blood glucose
GLUT-4	glucose transporter type 4
WC	waist circumference
EPOC	excess post-exercise oxygen consumption
eNOS	endothelial nitric oxide synthase
NO	nitric oxide
ROS	reactive oxygen species
LVEF	left ventricular ejection fraction
